# Active Seizures on Arrival at the Hospital During Ambulance Transfers in Children: A Five-Year Record Review

**DOI:** 10.7759/cureus.102705

**Published:** 2026-01-31

**Authors:** Masahiko Kimura, Hiroshi Ito, Ryuuji Sota, Takeshi Taketani

**Affiliations:** 1 Pediatrics Department, Kimura Children and Family Clinic, Izumo, JPN; 2 Shimane Izumo Fire Department, Izumo Fire Station, Izumo, JPN; 3 Pediatrics Department, Shimane University Faculty of Medicine, Izumo, JPN

**Keywords:** afebrile seizures, ambulance transfer, febrile seizures, incidence, pre-hospital treatment, status epilepticus

## Abstract

Introduction

Seizure disorders are common in childhood, and some patients develop prolonged seizures or status epilepticus (SE). It remains unknown what proportion of children experience continuous seizures upon hospital arrival after ambulance transfer. In Japan, prehospital treatment using antiseizure medications by the emergency medical service (EMS) is prohibited. Clarifying the natural course of seizures during ambulance transfer may help develop policies to prevent prolonged seizures in children.

Methods

We reviewed the EMS records of the Izumo Fire Department, Shimane, Japan, from January 1, 2018, to December 31, 2022, for all cases of seizures in children aged six years or under, focusing on seizure continuation during ambulance transfer. The Izumo Fire Department covers areas around Izumo City, with a total population of approximately 175,000.

Results

Out of 1,164 emergency transfers, 667 were seizure-related (494 children), with febrile seizures comprising 558 cases (84%). Active seizures were noted at the emergency call site in 19% (n = 126) of transfers upon ambulance arrival. Of these, 66% continued to seize until hospital arrival. Upon arrival at the hospital, seizures persisted in 97 transfers (15%), including 83 continuous and 14 recurrent seizure episodes. The study recorded 93 episodes of SE (14%). The mean incidence of first-time SE based on ambulance transfers was 118 per 100,000 children aged six years or under, ranging from 88 to 142.

Conclusions

A substantial number of children transferred by ambulance had active seizures upon hospital arrival. Immediate treatment is warranted at the emergency call site upon ambulance arrival.

## Introduction

Seizures are common neurological disorders in childhood, with febrile seizures being the most prevalent. In Japan, the prevalence of febrile seizures is approximately 8%-10% [1,2], which is two to three times higher than that in the United States and Europe (3%-4%) [3,4]. Our previous questionnaire-based population study reported a prevalence of 12.3% for febrile seizures, with the incidence of status epilepticus (SE) being 184 per 100,000 children aged 36 months or under [5]. The incidence of SE in Japan was three to five times higher than that in the United States and Europe [6,7]. The proportion of febrile seizures developing into SE was similar: 5.1% in the United States [8] and 4.5% in our previous study [5]. The prevalence of febrile seizures remains considerably higher in Japan. Febrile seizures are the most prevalent cause of SE both in Japan and London, with the values being higher in the former (46% and 63%) [9,10] than in the latter (32%) [7].

SE is the most common neurological emergency occurring during childhood [7]. Management guidelines for SE recommend that first-line therapy be administered within 5-10 minutes of seizure onset [11,12]. Therefore, pre-hospital treatment is necessary. However, in Japan, the administration of antiseizure medications by the emergency medical service (EMS) is currently prohibited. As a result, ambulance teams provide care to children with continuous seizures without intervention by antiseizure medications. It remains unknown what proportion of children experience continuous seizures upon hospital arrival after ambulance transfer. This study aimed to determine the proportion of children with active seizures on hospital arrival and to estimate the incidence of SE based on ambulance transfers. Clarifying these clinical questions can help develop policies to prevent prolonged seizures in children.

## Materials and methods

Study design and setting

This study retrospectively reviewed the EMS records of the Izumo Fire Department, Shimane, Japan, for all reported seizure-related ambulance transfers in children aged six years or under, between January 1, 2018, and December 31, 2022. The Izumo Fire Department covers areas around Izumo City, with a total population of approximately 175,000.

Data collection

The variables included age (date of birth), sex, the presumed time of seizure onset, the time of the emergency call, and the times of EMS arrival at both the emergency call site and the hospital. When duplicate dates of birth were identified, additional verification procedures, such as confirmation of their address, were performed to avoid misidentification of children. The seizure onset time was self-reported by caregivers. Clinical information, such as continuation of seizure and physical findings at the emergency call site, during transportation, and upon arrival at the hospital emergency department, was also obtained. Data regarding clinical severity and diagnosis, as assessed by the EMS and hospital emergency teams, were also collected.

Definitions

Active Seizure

An active seizure was defined as a documented convulsion, which was either focal or generalized, and tonic, clonic, or tonic-clonic, in the EMS records.

State of Children

The state of children was categorized into three groups: “clear,” “unclear,” and “seizure ongoing.” If children cried, opened their eyes, spoke, or showed stable respiration, the state of children was defined as “clear.” If children appeared atonic, somnolent, or without eye contact, the state of children was defined as “unclear.” If children had an apparent convulsive seizure, it was defined as “seizure ongoing.”

Clinical Severity

Clinical severity was categorized into three groups: “mild,” “moderate,” and “severe.” “Mild” clinical severity indicated that a child was not hospitalized and was discharged. Children categorized as “moderate” and “severe” were hospitalized. Children who required more than usual therapeutic interventions - such as close monitoring, respirator usage, or intensive care unit admission - were categorized as “severe.”

Definition of febrile seizure

“A febrile seizure” was defined as a seizure associated with a body temperature of 38℃ or higher. Typically, seizures occurring in children below the age of six months were excluded from a febrile seizure diagnosis [13,14]. However, in this study, fever-related seizures with a temperature of ≥38℃ in children aged below six months or under were also considered febrile seizures. In some instances, the hospital emergency team diagnosed febrile seizures even when the temperature recorded by EMS was <38℃ during transfer. Children presenting with a fever <38℃ were considered to have experienced afebrile seizures, including epileptic seizures, based on the hospital diagnosis. Clinical characteristics of children with febrile and afebrile seizures were compared. Some children had both febrile and afebrile seizures. In such cases, each seizure was categorized as either a febrile or an afebrile seizure, according to the definition, independently.

Definition of SE and the calculation of the incidence

SE was defined as seizures lasting ≥30 minutes [13]. Intermittent SE was defined as intermittent seizures lasting 30 minutes or more, without recovery of consciousness [13]. In this study, intermittent SE was defined as a condition in which the seizure subsided temporarily but recurred and continued until hospital arrival, with the child exhibiting an “unclear” state throughout transportation. To determine the incidence of SE, only the first-ever episode was counted for each child, even if multiple episodes of SE occurred. The incidence of SE due to febrile seizures was also calculated. The population aged six years or under in Izumo City was obtained annually, as of March 31, between 2018 and 2022. Both annual and average incidence rates over the study period, from January 1, 2018, to December 31, 2022, were calculated. Children who were not residents of Izumo were excluded. 

Outcomes

The primary outcome was the proportion of children who continued to experience seizures upon arrival at the hospital. The secondary outcome was to determine the incidence of SE, based on ambulance transfers in Izumo.

Ethical statement

The study protocol was approved by the Ethics Committee of Shimane University School of Medicine (KS20221117-2) and complied with the principles of the Declaration of Helsinki. The requirement for informed consent was waived, based on the opt-out principle.

Statistical methods

Descriptive statistical analyses were performed using Microsoft Excel, version 14 (Microsoft® Corp., Redmond, WA, USA). All data are presented as median and interquartile range (IQR) for skewness. Comparisons between febrile and afebrile seizure groups were conducted using Fisher’s exact test for categorical data and the Mann-Whitney U test for continuous data. Differences were considered significant at p < 0.05 (two-tailed). EZR, ver. 1.55 (Saitama Medical Center, Jichi Medical University, Saitama, Japan) [15], a graphical user interface for R ver. 4.0.3 (The R Foundation for Statistical Computing, Vienna, Austria) was used for statistical analysis.

## Results

Transferred children and seizure classification

From January 1, 2018, to December 31, 2022, a total of 1,164 emergency transfers involved children aged six years or under. Among these, 667 transfers (57%), representing 494 individual children, were attributed to seizure-related conditions (Table 1).

**Table 1:** Emergency transfers for seizure-related events among children aged ≤6 years, 2018-2022 *Includes afebrile seizures (unprovoked seizures and epilepsy) and breath-holding spells.

Year	Total transfer	Transfers for seizures	Febrile seizures	Non-febrile seizures*
2018	264	170	148	22
2019	241	131	106	25
2020	173	94	81	13
2021	230	123	101	22
2022	256	149	122	27
Total	1,164	667	558	109

Of all seizure transfers, 558 were classified as febrile seizures, while 109 were categorized as non-febrile events, including afebrile seizures (unprovoked seizures and epilepsy) and breath-holding spells. Among the 494 children transferred for seizure-related events, 258 (52%) were male. Regarding transfer frequency, 399 children were transferred once, 61 were transferred twice, 24 were transferred three times, six were transferred four times, and one child each experienced 6, 8, 11, and 25 transfers (Table 2).

**Table 2:** Summary of transferred children n: number of children

Category	Details	n
Total children transferred for seizures		494
Male children		258 (52%)
Frequency of transfers per child	1 transfer	399
	2 transfers	61
	3 transfers	24
	4 transfers	6
	6 transfers	1
	8 transfers	1
	11 transfers	1
	25 transfers	1

Of the 667 total transfers, 16 were inter-facility transports, involving either hospital-to-hospital or clinic-to-hospital transfers. The median age at the time of transfer was 23 months (IQR: 16-36 months), calculated across all transfer events, including multiple transfers per child. Body temperature was documented in 652 of the 667 transfers. Among these, 516 children had a recorded temperature ≥38.0°C during transfer. Of the 15 transfers without temperature data, the hospital emergency team diagnosed 11 as febrile seizures and four as afebrile seizures. Additionally, among 31 transfers with measured temperatures <38.0°C, children were clinically diagnosed with febrile seizures. Taken together, 558 of the 667 transfers (84%) were classified as febrile seizures: 516 with temperatures ≥38.0°C, 31 with temperatures <38.0°C, and 11 without temperature data but with a clinical diagnosis of febrile seizures (Table 3).

**Table 3:** Summary of transfers: seizure type, temperature classification, and demographic characteristics n: number of transfer

Category	Details	n
Total seizure-related transfers		667
Inter-facility transfers	Hospital-hospital or clinic-hospital	16
Median age at transfer	23 months (IQR 16-36)	
Temperature data available	Transfers with temperature recorded	652
≥38.0°C	Clinical fever	516
<38.0°C	Clinically diagnosed as febrile seizures	31
No temperature data	Febrile seizures	11
	Afebrile seizures	4
Total febrile seizures	Clinical + measured	558 (84%)
	Temp ≥38.0°C	516
	Temp <38.0°C	31
	No temperature data	11
Age distribution in febrile seizures	<6 months	2 (0 and 4 months)
	6 months to 5 years	518
	6 years	38
Total non-febrile seizures	Afebrile/epilepsy + breath-holding spells	109
	Afebrile seizures/epilepsy	106
	Breath-holding spells	3
Children with both febrile and afebrile seizures		11 children
Children with both afebrile seizures and breath-holding spells		1 child

Within the febrile seizure group (n = 558), two children were younger than six months of age (zero and four months), and 38 were six years old. Among the remaining 109 transfers, 106 were attributed to afebrile seizures, including epilepsy, and three were due to breath-holding spells. Eleven children experienced both febrile and afebrile seizure events. One child had both an afebrile seizure and a breath-holding spell (Table 3).

Active seizures at the emergency call site and hospital, recurrent seizures, and the incidence of SE

**Figure 1:**
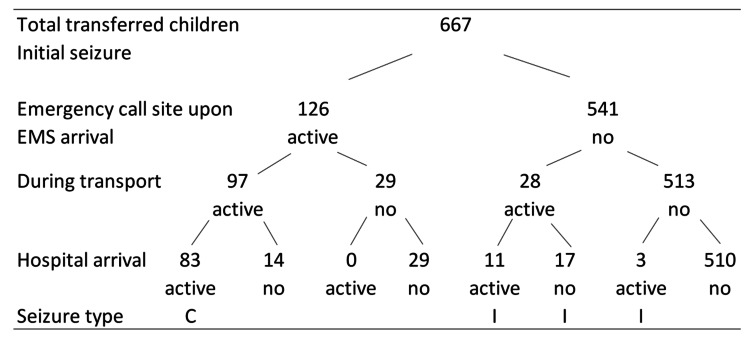
Number of children with or without seizures at each stage of the prehospital and hospital time course At the emergency call site, 126 children (19%) exhibited active seizures when the ambulance team arrived. On hospital arrival, 97 children (15%) were still experiencing active seizures, of whom 83 had continuous seizures from onset. Thus, 66% (83/126) of children who were seizing at the emergency call site continued to have active seizures upon arrival at the hospital. active: active seizures; no: no seizures; C: continuous seizures; I: intermittent seizures

In total, children in 126 (19%) and 97 (15%) transfers were seizing when the ambulance team arrived at the emergency call site and on arrival at the hospital, respectively. Of the 97 children, 83 had continuous seizures from onset, and 14 had recurrent seizures (Figure 1).

The median time from the emergency call to ambulance arrival at the emergency call site was 7 minutes (IQR: 6-9 minutes), and from the emergency call to hospital arrival was 26 minutes (IQR: 21-30 minutes). Among the 83 children who had continuous seizures, the seizure onset time was noted in 79, with a median seizure duration of 31 minutes (IQR: 27-36 minutes). When children had a seizure at the emergency call site upon ambulance arrival, 66% (83/126) continued seizing upon hospital arrival (Table 4).

**Table 4:** Time course of the transfer

	n	Emergency call to the emergency call site	Emergency call to the hospital	Number of transfers presumed seizure onset time obtained	Seizure onset to the emergency call site	Seizure onset to the hospital
All transfers	667	7 (6-9) minutes	26 (21-30) minutes	638	13 (10-17) minutes	32 (28-38) minutes
Continuous seizure from onset to the hospital	83	7 (6-9) minutes	23 (20-28) minutes	79	14 (11-18) minutes	31 (27-36) minutes

During the transfer, 31 children (4.6%) experienced recurrent seizures. Among these, 17 had seizures that recurred during transportation and subsided upon arrival at the hospital. Meanwhile, 11 children continued seizing upon arrival, and three experienced recurrences only upon arrival at the hospital. Of these 14 children who were seizing at the hospital, 10 exhibited an “unclear” state between seizure episodes. These 10 children were considered likely to have intermittent SE.

Regarding the incidence of SE, seven children experienced multiple episodes, ranging from 2 to 15 occurrences, and only the initial event of each child was counted. The mean incidences of SE from 2018 to 2022 were 118, with a range of 88-142 per 100,000 children aged six years and younger for all seizures, and 85, ranging from 58 to 119, for febrile seizures among children transported by ambulance (Table 5).

**Table 5:** Incidence of status epilepticus among children aged ≤6 years in Izumo from 2018 to 2022 Note: Incidence calculated per 100,000 children aged ≤6 years. SE: status epilepticus

Year	Status epilepticus (intermittent SE)	SE in children living in Izumo (intermittent SE)		First-time SE in Izumo (febrile SE)	Population ≤ 6 years in Izumo	Incidence of SE ≤ 6 years (incidence of febrile SE)
2018	24 (1)	24 (1)	14 (13)		10,933	128 (119)
2019	21 (2)	21 (2)	14 (8)		10,861	130 (74)
2020	13 (0)	13 (0)	9 (6)		10,278	88 (58)
2021	21 (5)	18 (4)	15 (11)		10,562	142 (104)
2022	14 (2)	14 (2)	11 (7)		10,562	104 (66)
2018-2022	93 (10)	90 (9)	63 (45)		53,196 (person-years)	118 (85) (mean)

Comparison of febrile and afebrile seizures

When comparing children with febrile seizures to those with afebrile seizures, including epilepsy, children with afebrile seizures were older and more likely to be actively seizing upon ambulance team arrival at both the emergency call site and the hospital (Table 6).

**Table 6:** Comparison of febrile and afebrile seizures *Mann-Whitney’s U test, ** Fisher’s exact test. Note: ^a ^Percentage calculated based on unique patients, not total transfers. ^b ^Afebrile Seizures group included unprovoked seizures and epilepsy, excluding three transfers of breath-holding spell. ^c ^Total for clinical severity of afebrile seizures sums to 105; one transfer had missing severity data. Eleven children had both febrile and afebrile seizures. IQR: interquartile range; SE: status epilepticus; n: number of transfer

		Febrile seizures	Afebrile seizures^b^	p
Ambulance transfer, n		558	106	
Age at transfer, months, median (IQR)		21 (16-34)	46 (31-66)	<0.001*
Male sex (transfers), n (%)		304 (54%)	39 (37%)	<0.001**
Unique patients				
Total children, n		452	51	-
Boys, n (%)^a^		242 (54%)	23 (45%)	0.46**
Active seizure at scene, n (%)		83 (15%)	43 (40%)	<0.001**
Active seizure on hospital arrival, n (%)		61 (11%)	36 (34%)	<0.001**
Continuous seizure (scene to hospital), n (%)		51 (9%)	32 (30%)	<0.001**
Total SE (including intermittent), n (%)		57 (10%)	36 (34%)	<0.001**
Persistence of seizure (scene to hospital)	51/83 (61%)		32/43 (74%)	0.169**
Clinical severity, n (%)^c^				<0.001*
Mild	375 (67%)		37	
Moderate	159 (28%)		48	
Severe	24 (4%)		20	

One data point on clinical severity from the afebrile group was not obtained. Clinical severity was higher among children with afebrile seizures than among those with febrile seizures. Children with afebrile seizures also experienced a greater number of recurrent seizure events, as reflected in the frequency of ambulance transfers (Table 7).

**Table 7:** Number of ambulance transfer per child, comparing febrile and afebrile seizures Mann-Whitney’s U test, p < 0.01; n: number of children. Eleven children experienced both febrile and afebrile seizures. Three transfers due to breath-holding spells were excluded.

Number of transfer	Febrile seizures (n)	Afebrile seizures (n)
1	375 (83%)	33 (65%)
2	51 (12%)	12 (24%)
3	23 (5%)	0
4	3 (0.7%)	3 (6%)
5	0	0
6	0	0
7	0	0
8	0	2 (4%)
21	0	1 (2%)
Total number of children	452	51

Notably, three children with afebrile seizures underwent more than eight transfer episodes. A review of these three children revealed that one child was diagnosed with Dravet syndrome, which accounted for 25 transfer events, including 15 episodes of SE. Among these transfers, febrile seizures occurred during four episodes, whereas afebrile seizures occurred during 21 episodes.

As for febrile seizures, which were five times as common as afebrile seizures in this study, children in 83 (15%) and 61 (11%) transfers were seizing when the ambulance team arrived at the emergency call site and on arrival at the hospital, respectively, and 61% (51/83) of children who had a seizure upon ambulance arrival continued to have active seizures upon reaching the hospital (Table 6). 

## Discussion

The study evaluated ambulance transfer due to seizure disorders in children aged 6 or younger by reviewing EMS records in Izumo, Japan. Seizure disorders were the most common causes of ambulance transfer in this age group. The results showed that 19% of children were actively seizing when the ambulance team arrived at the emergency call site, while 15% were seizing on arrival at the hospital. In this study, SE was defined according to “Guidelines for epidemiological studies on epilepsy” [13]; however, more practically, the International League Against Epilepsy issued a new guideline [16]. The median time from the emergency call to ambulance arrival at the emergency call site was seven minutes. According to the new guideline, since these seizures lasted more than five minutes, these children met the t1 definition of SE, indicating the point at which treatment should begin to prevent prolonged seizures [16]. Furthermore, in this study, 66% of children who were seizing at the emergency call site continued to experience a seizure upon arrival at the hospital. The median time from seizure onset to hospital arrival was 32 minutes. In practice, even more time may be required to terminate seizures. The prolonged seizures from onset to hospital arrival in this study met the definition of t2 of SE, which indicates when long-term consequences may appear [16]. Therefore, children who have an active seizure when the ambulance team arrives at an emergency call site should be treated immediately.

In this study, seizures recurred during transfer in 31 children (4.6%). Among them, 10 experienced recurrent seizures upon arrival at the hospital without regaining consciousness from the initial seizure and were diagnosed as having intermittent SE [13]. Therefore, 83 cases of continuous and 10 cases (10/93, 11%) of intermittent SE were identified. In previous studies, intermittent seizures represented 23% of prolonged febrile seizures [17] and 42%-58% [7,18] of all SE. Although the low frequency of intermittent SE in this study remains unexplained, the ambulance team documented the presence of seizures each time on arrival at the emergency call site, during transportation, and on arrival at the hospital; therefore, the diagnosis of intermittent SE was considered reliable.

Active seizures on the EMS team’s arrival at the emergency call site and on arrival at the hospital were further evaluated and divided into febrile seizures and afebrile seizures. Children with afebrile seizures were generally older, underwent multiple transfers, and were more likely to experience prolonged seizures and SE. The clinical severity of children with afebrile seizures was also increased. However, certain epileptic syndromes characterized by repeated SE, particularly Dravet syndrome, as seen in this study, may have contributed to the increased severity in the afebrile seizure group.

In this study, the mean incidence of first-ever SE was 118 (88-142) per 100,000 children aged six years or younger. The subjects included children who were residents of Izumo and were transported by ambulance. In this study, febrile SE accounted for 71% of the cases. The incidence of SE per 100,000 children aged four years or younger was 57.2 for all SE and 16.9 for febrile SE in Rochester, USA [6], and 33.5 and 13.8, respectively, in North London, UK [7]. In Okayama, Japan, the incidence was 65.8 for all SE and 35.1 for febrile SE per 100,000 children aged six years or younger [9]. The incidence of SE in our study was higher than that not only in the United States and Europe [6,7] but also in Japan [9]. We speculated that the high incidence of SE was due to a high prevalence of febrile seizures [5]. This study also showed a high proportion (71%) of febrile SE among total SE cases. Our previous study, using questionnaires from caregivers, also showed a high prevalence of febrile seizures (12.3%) and a high incidence of febrile SE, at 184 per 100,000 children aged three years or younger, in the same area [5]. Regarding the higher incidence in our study compared with previous studies, case ascertainment in previous studies was hospital-based in Okayama [9] and based on a regional database in Rochester and London [6,7]. Our study included all children with SE who were transported by ambulance during the study period. Moreover, the underlying population of this study was much smaller than those of previous studies [6,7,9]; therefore, it is difficult to directly compare the current study with them. However, we believe that the incidence of SE may be higher than previously reported. In this study, the incidence of SE included only children brought by ambulance, and this rate would be higher if children brought by caregivers were also considered. Our previous study showed that only 32% of children were transported by ambulance [5]. Furthermore, this transport rate was considerably lower than that reported in Israel (77%) [17], the United States (90%) [19], and Tokyo (75%) [20]. This is an issue related to pre-hospital treatment.

Early termination of seizures is essential for the treatment of SE. Alldredge et al. reported that seizure termination upon hospital arrival was 59.1%, 42.6%, and 21.1% in the intravenous lorazepam, diazepam, and placebo administration groups, respectively, when administered by paramedics as pre-hospital treatment [21]. Patients were treated if they had convulsive seizures lasting ≥5 minutes. The rates of respiratory or circulatory complications were not statistically different among the three groups. Using a similar protocol, the proportion of seizure termination upon arrival at the hospital was 73.4% and 63.4% in the intramuscular midazolam administration group and intravenous lorazepam group, respectively [22]. According to these results, our study showed that the rate of spontaneous seizure termination upon arrival at the hospital in children who were seizing at the emergency call site was 34% ((1-83/126) × 100). If pre-hospital treatment had been performed at the emergency call site, seizures would have terminated upon arrival at the hospital in an additional 30% of children in this study. In one case series, 45% of ongoing prolonged febrile seizures were terminated by antiseizure drugs while being transferred by ambulance [17]. Failure to administer pre-hospital treatment was associated with a risk of seizure prolongation ≥60 minutes, with an odds ratio of 2.4 [23].

This study showed a high proportion of SE in febrile seizures (10%) and a high incidence of febrile SE. In particular, children with prolonged febrile seizures are at risk for two potential outcomes. First, acute encephalopathy may occur. In Japan, acute encephalopathy with biphasic seizures and late reduced diffusion (AESD), or acute encephalopathy with febrile convulsive SE, was the most prevalent acute encephalopathy [24]. Ichinose et al. reported that acute encephalopathy occurred more frequently with longer seizure duration in febrile seizures, with rates of 4.3% and 7.1% in patients with seizures lasting 20 minutes and 40 minutes, respectively [25]. Therefore, one preventive measure against encephalopathy is to treat children with ongoing febrile seizures promptly. Second, febrile SE caused acute diffusion-weighted imaging hippocampal hyperintensity in 27% of children. This finding is related to mesial temporal lobe epilepsy [26]. These findings were observed in children with seizures lasting a median of 70 minutes.

Although pre-hospital treatment with antiseizure medications through EMS is not permitted in Japan, since December 2020, buccal midazolam, which is one of the treatment alternatives in pre-hospital settings [12], has become available for the treatment of SE out of hospital by caregivers. In this study, repeated episodes of SE, especially in the afebrile seizure group, could have been prevented if buccal midazolam had been available at home. Practically, buccal midazolam at home was not widely available during the study period. In the case of febrile seizures, 85% of children with either prolonged febrile seizures or SE do not have a prior febrile seizure [5,27]; therefore, neither buccal midazolam at home nor preventive antiseizure medications, such as diazepam suppositories, can be prepared [14]. To mitigate SE in pediatric seizure disorders, intervention for febrile seizures is the most important issue because of their high prevalence and the aforementioned relationship with encephalopathy and epilepsy. For this purpose, immediate treatment by the EMS team is warranted.

This study has several limitations. First, it was not always possible to determine whether the seizures were active. In this study, convulsions were used as the key indicators of active seizures. Consequently, non-convulsive seizures or seizures with subtle clinical manifestations, such as eye deviation, may have gone undetected. Lack of information, such as EEG data from hospital records, prevents differentiation between prolonged convulsive seizures and other motor events [28,29]. However, close monitoring of the entire clinical course, from the emergency call site to the hospital, by the ambulance team can minimize misinterpretations. A study on identifying convulsive SE by ambulance emergency teams showed high specificity but low sensitivity; however, in pediatric patients, the sensitivity was high (88.0%) [30].

Second, although the incidence of SE was higher than that reported in previous studies [6,7,9], the underlying population of this study was much smaller. Findings from a single regional city, Izumo, cannot be generalized. However, the EMS covers all patients in need in the city. Records from five consecutive years, including the COVID-19 outbreak period, showed a consistently high incidence of SE. Moreover, our previous study, using questionnaires from caregivers, also showed a high incidence of febrile SE, at 184 per 100,000 children aged three years or younger, in the same area [5].

Third, in comparisons between febrile and afebrile seizures, the afebrile seizure group had a higher frequency of ambulance transfers per child and SE. However, some children with multiple transfers, including those with Dravet syndrome, could have influenced the clinical severity of the afebrile seizure group in this small background population.

Fourth, we did not have detailed information pertaining to diagnosis, therapy, and prognosis from hospital records. We reviewed EMS records without validation from hospital records and relied on diagnoses made by hospital emergency teams, even for some children presenting with a fever <38°C who were classified as having febrile seizures. Moreover, we did not know the outcomes of transferred children and could not clarify the current burden without intervention by the EMS team. A prospective study involving both ambulance and hospital teams would be desirable to obtain precise outcome data in pediatric ambulance transfers.

## Conclusions

In this five-year, population-based study of children aged six years or younger who were transported by ambulance for seizures, a substantial proportion continued to experience active seizure activity on arrival at the hospital. Two-thirds of the children who were seizing at the emergency call site when the ambulance team arrived remained in seizure upon arrival at the hospital, and the incidence of status epilepticus was high. These findings highlight a critical period in the prehospital phase during which timely intervention may prevent ongoing seizures and progression to status epilepticus.
